# Effect of Steel Slag Content on the Performance and Hydration Mechanism of Phosphogypsum–Steel Slag–Fly Ash All-Solid-Waste Binders

**DOI:** 10.3390/ma19112249

**Published:** 2026-05-26

**Authors:** Di Liu, Yazhou Wang, Binbin Zhang, Yu Ma

**Affiliations:** College of Smart City Engineering, Shangqiu Normal University, Shangqiu 476000, China; sqnuliudi@sqnu.edu.cn (D.L.); myshangqiu6@163.com (Y.M.)

**Keywords:** phosphogypsum, steel slag, fly ash, hydration reaction, pore structure

## Abstract

To promote the synergistic utilization of phosphogypsum (PG), steel slag (SS), and fly ash (FA), a ternary all-solid-waste binder, namely PG-SS-FA cementitious material (PSA), was prepared. The effects of SS content on workability, setting behavior, mechanical properties, hydration products, pore structure, and microstructure were systematically investigated. The results showed that increasing SS content continuously reduced the fluidity of PSA, while the setting time first shortened and then increased. The fastest setting was observed at 40% SS, with initial and final setting times of 126 and 321 min, respectively. Increasing SS from 20% to 40% enhanced the hydration reaction, promoted the formation of AFt and C-(A)-S-H gel, reduced residual unreacted phases, and refined the pore structure, resulting in the highest compressive and flexural strengths for M40. However, further increasing SS to 60% and 80% reduced the fly ash proportion and limited the sustained supply of reactive Si/Al species, despite increasing Ca^2+^ availability and alkalinity, thereby restricting later-age gel accumulation and pore refinement and ultimately weakening mechanical performance. Overall, the performance evolution of PSA is governed by the coupled effects of alkali/Ca supply from SS, sulfate supply from PG, and reactive Si/Al supply from FA. The optimal performance at 40% SS is attributed to the synergistic construction of an AFt framework and continuous pore filling by C-(A)-S-H gel.

## 1. Introduction

Ordinary Portland cement (OPC) is the most widely used cementitious material in construction engineering. However, OPC production is a major contributor to global carbon dioxide emissions, accounting for approximately 5–7% of total anthropogenic CO_2_ emissions [1,2]. With cement production projected to be 45% higher than current levels by 2050 [3], the environmental pressure associated with OPC manufacturing continues to intensify. Simultaneously, the rapid advancement of industrialization has led to the generation of substantial volumes of industrial solid waste, including fly ash, steel slag, furnace slag, red mud, and phosphogypsum [4,5]. In China alone, approximately 3.3 billion tons of industrial solid waste are produced annually, with historical stockpiles exceeding 60 billion tons, covering more than 2 million hectares of land and posing significant ecological and safety hazards [6]. Therefore, developing sustainable alternatives to OPC while achieving large-scale resource utilization of industrial solid waste has become an urgent priority.

Utilizing industrial solid waste to produce cementitious materials represents a crucial pathway for both waste valorization and carbon emission reduction [7,8,9]. The most prevalent current approach involves incorporating these wastes as supplementary cementitious materials (SCMs) to partially replace OPC [10,11,12]. However, the inherently low reactivity of most industrial solid wastes severely restricts their substitution ratio and consequently limits overall carbon reduction efficiency [13,14]. To overcome this limitation, researchers have increasingly focused on developing novel low-carbon cementitious systems based on synergistic interactions among multiple solid wastes [15,16]. Among the various industrial wastes, steel slag (SS) contains calcium silicate phases with hydraulic activity capable of spontaneously reacting with water to form C–S–H gel and Ca(OH)_2_, thereby generating an alkaline environment [17]. Fly ash (FA) is rich in reactive silica and alumina, which can undergo pozzolanic reactions under alkaline conditions [18]. Phosphogypsum (PG), primarily composed of CaSO_4_·0.5H_2_O, can serve as a sulfate activator [19]. The combination of these three wastes is expected to establish a “alkali activation–sulfate synergy–pozzolanic reaction” mechanism: the alkali from SS hydration activates FA, while sulfate from PG promotes ettringite (AFt) formation, together accelerating hydration and optimizing pore structure. Recent studies have explored multi-component solid-waste-based cementitious systems, including ternary and quaternary binders. In this context, a ternary solid-waste-based system refers to a binder mainly composed of three industrial solid wastes, whereas a quaternary system contains four solid waste components. These systems are generally designed by combining wastes with complementary chemical functions, such as alkalinity and Ca supply from steel slag, reactive Si/Al supply from fly ash or slag, sulfate supply from phosphogypsum, and additional pore-filling or gel-forming contributions from other metallurgical wastes. For example, alkali-activated steel slag–fly ash composites [20,21], steel slag–phosphogypsum cementitious materials [22], and low-carbon binders incorporating multiple metallurgical slags [23,24] have been investigated, demonstrating the feasibility of using synergistic interactions among different solid wastes to develop low-carbon cementitious materials. In these systems, the term “synergistic approaches” refers to the deliberate combination of different solid wastes with complementary chemical functions. For the PG-SS-FA system, SS hydration and dissolution provide OH- and Ca^2+^, thereby creating an alkaline environment for FA activation; FA supplies reactive Si and Al species for C-(A)-S-H gel formation; and PG provides SO_4_^2−^ and additional Ca^2+^ to promote AFt formation. The AFt crystals can construct a skeletal framework, while C-(A)-S-H gel progressively fills pores and binds particles together. Thus, the synergy is reflected not only in the coupled reactions of alkaline activation, sulfate activation, and pozzolanic reaction but also in the combined microstructural effects of hydration product intergrowth, pore filling, and matrix densification.

Despite these advances, several critical knowledge gaps remain regarding the SS-FA-PG ternary system. First, the regulatory mechanism of SS content on the setting and hardening behavior of the ternary system remains insufficiently understood, particularly the “encapsulation effect” whereby excessive SS leads to rapid early reaction kinetics that coat FA particle surfaces with hydration products, inhibiting subsequent dissolution and sustained reaction [25,26]. Second, the evolution of hydration products and their relationship with pore structure refinement, especially the fraction of large pores (>100 nm), has not been systematically characterized using integrated microscopic techniques. Third, existing studies have largely focused on empirical performance characterization, with limited development of predictive models that can guide mix proportion optimization for practical engineering applications. Furthermore, while prediction models for mechanical properties based on response surface methodology have been developed for quaternary waste-based systems [23], such models are lacking for the SS-FA-PG ternary system, particularly those incorporating pore structure parameters as key predictors.

Although steel slag and steel-related wastes have been widely investigated in cement and concrete, most previous studies have used them as aggregates, supplementary cementitious materials, conductive fillers, or functional components. For example, steel wire rope and steel powder wastes have been incorporated into conductive concrete to improve electrical conductivity and deicing performance [27], while other studies have evaluated the sustainability of conductive concrete in pavement or bridge applications [28]. These studies mainly focused on the functional performance of concrete, such as electrical conductivity, deicing efficiency, compressive strength, or sustainability assessment. In contrast, the present study focuses on a phosphogypsum–steel slag–fly ash ternary all-solid-waste binder in which steel slag acts not only as a solid waste component but also as a source of alkalinity and reactive Ca for activating fly ash and promoting hydration reactions. Therefore, the novelty of this work is not simply the use of steel slag in concrete but the construction of a ternary all-solid-waste cementitious system and the clarification of the coupled reaction mechanism among steel slag, phosphogypsum, and fly ash. Based on the above analysis, this study systematically investigates the SS-FA-PG ternary cementitious system with varying SS dosages (20–80%). The setting time, fluidity, compressive strength, and flexural strength of the composite binder are comprehensively evaluated. The hydration products, pore structure characteristics, and microstructural evolution are characterized using X-ray diffraction (XRD), thermogravimetric analysis (TG-DTG), scanning electron microscopy (SEM), Fourier transform infrared spectroscopy (FTIR), and mercury intrusion porosimetry (MIP). The underlying mechanisms governing the influence of SS content on reaction degree and microstructural densification are elucidated.

## 2. Materials and Methods

### 2.1. Raw Materials

The main raw materials for preparing SS-FA-PG mortar included SS, FA, PG, sand, and water. SS, FA, and PG were collected from Weifang Special Steel Group Co. (Weifang, China), Weifang Huadian Power Plant (Weifang, China), and Shandong Red Sun Chemical Co. (Jinan, China), respectively. The sand used was standard sand with SiO_2_ content greater than 96%, loss on ignition below 0.40%, and clay content below 0.20%, provided by Xiamen Aisiou Co. (Xiamen, China). The mixing water was tap water supplied in Shangqiu city, and the quality meets the requirements as per the Chinese standard JGJ63-2006 [29].

**Figure 1:**
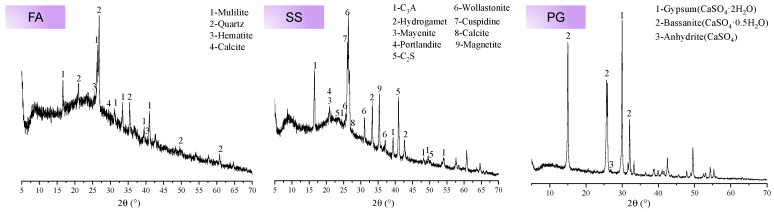
X-ray diffraction patterns of fly ash, steel slag, and phosphogypsum.

The chemical compositions of SS, FA, and PG were achieved using the X-ray fluorescence (XRF) technique. The chemical compositions of SS, FA, and PG are given in Table 1 and compared to those of OPC. As shown in Table 1, the total content of SiO_2_, Al_2_O_3_, Fe_2_O_3_, and CaO in the chemical composition of SS, FA, PG, and OPC accounted for 90.5%, 91.1%, 42.4%, and 86.36%, respectively. Hence, the main chemical composition of SS and FA is similar to that of OPC, whereas PG differs significantly. The mineralogical compositions of SS, FA, and PG were analyzed using the X-ray diffraction (XRD) technique. The XRD patterns obtained from SS, FA, and PG are illustrated in Figure 1. Figure 1 indicates that the main phases of SS were calcium silicate, portlandite, and calcite, that those in FA were mullite, quartz, and hematite, and that the main phase in PG was CaSO_4_·0.5H_2_O. The phase composition of SS indicates its hydraulic nature, spontaneously reacting with water to form C-S-H gel and Ca(OH)_2_. Thus, hydration of SS generates alkaline species, creating the high-pH environment essential for FA activation.

**Figure 2:**
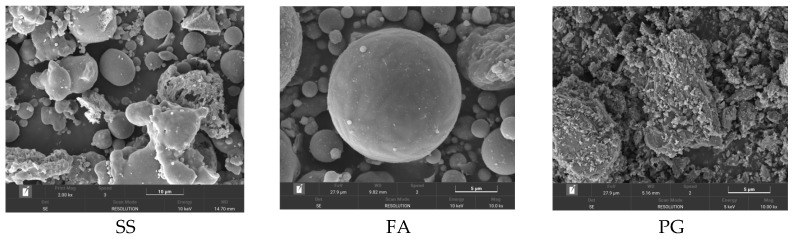
Scanning electron microscopy images of steel slag, fly ash, and phosphogypsum.

The SEM pictures taken from SS, FA, and PG are provided in Figure 2. Figure 2 displays the particle morphology of SS, which resembles that of OPC. SS particles exhibit well-defined edges and a relatively smooth surface. In contrast, PG particles feature irregular edges, with some fine particles adhering to the surface of larger grains. These fine particles may consist of impurities such as soluble phosphorus (H_3_PO_4_, CaHPO_4_), fluorides (NaF, CaF_2_), and organic phosphonic (as shown in Figure 2). The spherical particles observed in Figure 2 are FA, characterized by their smooth and rounded edges.

**Figure 3:**
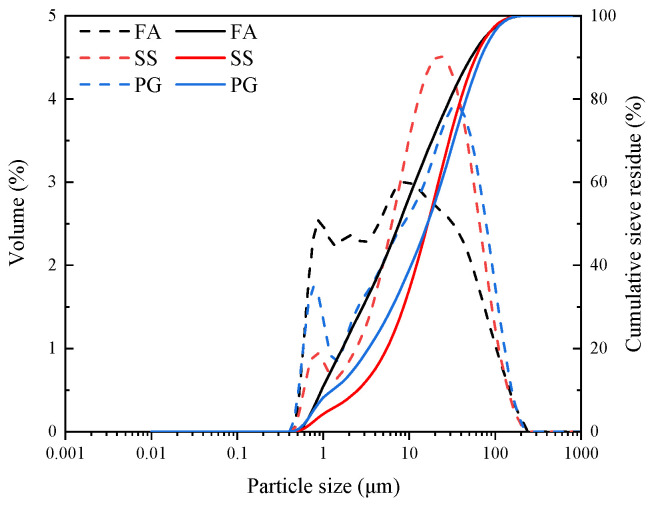
Particle size distribution of fly ash, steel slag, and phosphogypsum.

The particle size distributions of SS, FA, and PG were measured using a laser particle size analyzer, and the results are shown in Figure 3. As can be seen from Figure 3, the median particle sizes (D50) of SS, FA, and PG are 18.7 μm, 8.84 μm, and 18.5 μm, respectively. The fractions of particles smaller than 5 μm in SS, FA, and PG are 18.91%, 41.01%, and 26.63%, respectively. Thus, among the solid raw materials used in this study, FA contains a higher proportion of fine particles, with SS having the largest particle size, FA the smallest, and PG exhibiting an intermediate particle size.

### 2.2. Mixture Proportions and Preparation

Mortars were prepared using SS, FA, PG, water, and standard sand. The mixture proportions are shown in Table 2. As shown in Table 2, the designation “M” denotes mortar, with the numeral following “M” indicating the mass percentage of SS in the total binder (i.e., SS + FA + PG). For the four mortar groups detailed in Table 2, the water-to-binder ratio was set to be 0.45 and the binder-to-sand ratio was set to be 1:3. Furthermore, the mass ratio of PG to FA was always maintained at 1:2.

The required amount of PPG according to the mix proportion was weighed and placed into a mixer, followed by the addition of mixing water. The mixture was then mixed at a low speed for 60 s (note that the mixing time should not be excessively long to prevent premature setting and hardening of the paste within the mixing bowl). The mixed paste was cast into prepared molds, and the molds were subsequently placed on a vibration table to ensure compaction. Excess paste on the surface was scraped off with a spatula, the surface was smoothed, and the specimens were then cured indoors. Before use, a release agent was applied to the molds, and a piece of waterproof tape was adhered over the air holes at the bottom of each mold to reduce damage to the specimens caused by the air gun’s impact during demolding.

The dimensions of the specimens were 40 mm × 40 mm × 160 mm. Additionally, a series of paste specimens were prepared to investigate the hydration characteristics and microstructure of the SS-FA-PG composite binder. The proportions of SS, FA, and PG and the water-to-binder ratio in these pastes were identical to those in their corresponding mortar samples. After demolding, the mortar/paste specimens were cured in a standard curing room with a temperature of 20 ± 2 °C and relative humidity ≥ 95%.

### 2.3. Test Methods

#### 2.3.1. Setting Time

The initial and final setting times were determined using a Vicat apparatus, according to Chinese standard GB/T1346-2024 [31]. For each mixture, three paste samples were tested, and the average value was reported. The initial setting time was recorded at a needle penetration depth of 4 ± 1 mm above the mold’s bottom. The final setting time was defined as the point where the annular attachment left no visible indentation on the test block.

#### 2.3.2. Fluidity and Strength

This work also evaluated the applicability of the SS-FA-PG ternary system in terms of the fluidity of the prepared mortar. The fluidity of the 4 groups of mortar mentioned in Table 2 was tested by referring to the national standard of China GB/T2419-2005 [32]. For each mixture, the fluidity test was repeated three times, and the average value was reported. Compressive and flexural strength tests were conducted in accordance with Chinese standard GB/T 17671-2021 [33]. For each mixture and curing age, three 40 mm × 40 mm × 160 mm prism specimens were prepared. The three prisms were first used for flexural strength testing, and the six broken halves obtained after the flexural test were subsequently used for compressive strength testing. The average values were used as the final flexural and compressive strengths.

#### 2.3.3. Hydration Products and Reaction Degree

The mechanism for changes in the setting time, fluidity, and strength of SS-FA-PG composite binder with varying SS dosages was discussed according to the hydration process, the composition of the hydration products, and the microstructural characteristics of the SS-FA-PG paste. Based on this, the present work primarily examined the X-ray diffraction (XRD) patterns and scanning electron microscope (SEM) images of the SS-FA-PG paste at 28 days of aging and conducted thermogravimetric analysis (TGA) at 3, 7, and 28 days.

The SS-FA-PG paste samples were crushed and ground into fine powders. Subsequently, these fine powders were dried at 45 °C for 24 h and then used for XRD and TGA testing. In the XRD measurement, Cu-Kα radiation with a wavelength of 1.54 Å in the 5–70° of 2θ range was utilized, and the voltage was set to be 45 kV and the current was 40 mA. TGA was conducted in a helium atmosphere, with heating from room temperature to 900 °C at a rate of 5 °C/min. The samples for SEM testing were prepared by breaking the SS-FA-PG paste specimens into small flakes. Similarly, these flakes were dried at 45 °C for 24 h.

Fourier transform infrared spectroscopy (FTIR) was used to characterize the molecular structure and chemical composition of the samples. The 28 d paste specimens were manually ground into powder prior to testing, following the same pretreatment procedure as that used for TGA. FTIR spectra were collected using a Vertex 70 spectrometer equipped with a DTGS detector over the range of 4000–400 cm^−1^ at a resolution of 4 cm^−1^, with 32 scans for each measurement.

#### 2.3.4. Microstructure

Mercury intrusion porosimetry (MIP) was employed to characterize the pore structure and pore size distribution of the samples [34]. The 28 d paste samples were crushed into 0.5 cm pieces and dried at 40 °C for 24 h before testing. Measurements were conducted using a Quantachrome PM 60 porosimeter (Micromeritics, Norcross, GA, USA) at pressures ranging from 0.28 to 413.70 MPa, with the contact angle and mercury surface tension set to 140° and 480 mN/m, respectively.

## 3. Results

### 3.1. Setting Time

**Figure 4:**
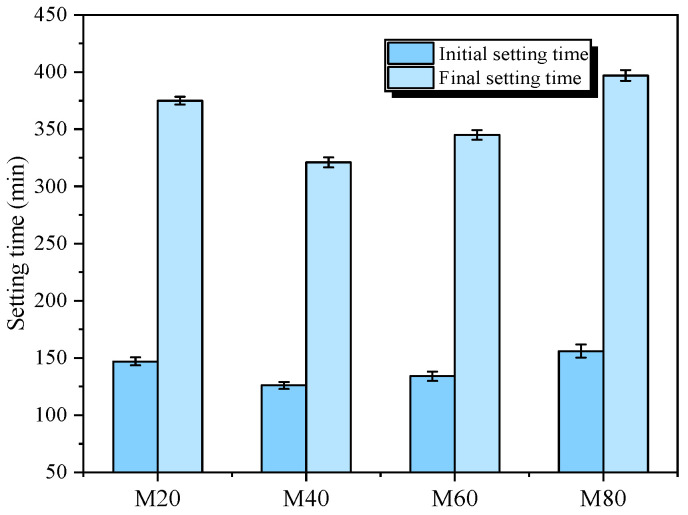
Setting time of PSA.

Figure 4 illustrates the variation of setting time in the PSA system with increasing SS content. It can be observed that as the SS content increases, both the initial and final setting times of PSA first shorten and then increase. When the SS content reaches 40%, the system exhibits the fastest setting time, with initial and final setting times of 126 min and 321 min, respectively. The primary reason for the shortened setting time is that SS provides a more strongly alkaline environment, which promotes precursor dissolution and hydration reactions, thereby accelerating the gel formation rate. This observation is consistent with findings reported in the literature [35]. On the other hand, when the SS content is further increased, the excessively rapid reaction kinetics tend to encapsulate the FA particle surfaces with quickly formed hydration products, inhibiting further dissolution and reaction. This, in turn, reduces the overall reaction degree and slows the setting and hardening processes of the system [35]. Therefore, as the SS content increases from 40% to 80%, the setting time of PSA is prolonged accordingly.

### 3.2. Fluidity

**Figure 5:**
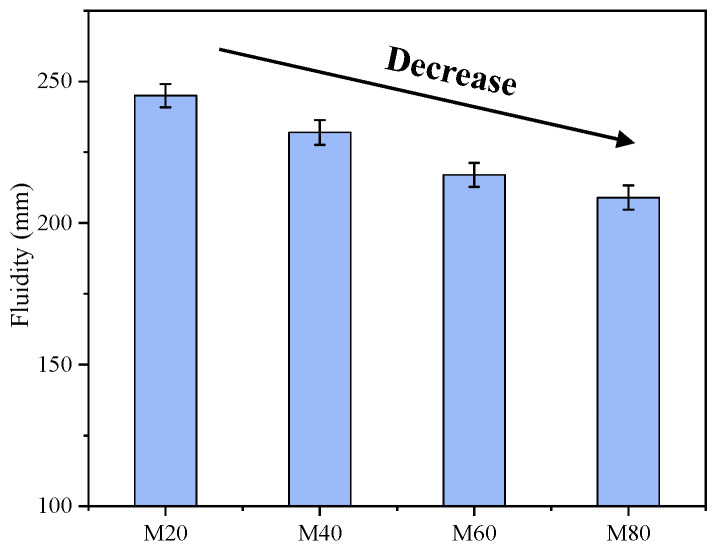
Fluidity of PSA.

Figure 5 shows that the fluidity of PSA decreased progressively with increasing SS content. As the SS content increased from 20% to 80%, the fluidity decreased from 245 mm to 209 mm, representing a reduction of 14.7%. This reduction can be attributed to the combined effects of enhanced reaction and the diminished contribution of FA to rheological improvement. Specifically, the higher SS content accelerated hydration, resulting in the formation of more flocculent reaction products that impaired fluidity. Meanwhile, the reduction in FA content weakened the ball-bearing effect of FA, further contributing to the loss of fluidity.

### 3.3. Compressive and Flexural Strength

**Figure 6:**
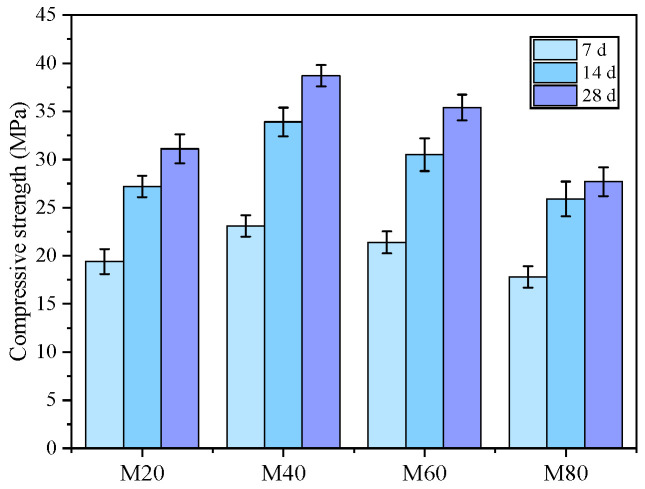
Compressive strength of phosphogypsum–steel slag–fly ash cementitious material.

Figure 6 presents the compressive strength development of specimens with varying SS contents at 7, 14, and 28 d. The 7-day compressive strength of all specimens exceeds 17 MPa, and the strength progressively increases with curing age, indicating that dissolution, polymerization, and gel formation reactions continuously occur within the system during curing, thereby promoting continuous densification of the internal structure. As the SS content increases, the compressive strength of PSA first increases and then decreases, reaching a maximum at 40% SS, with a 28-day compressive strength of 38.7 MPa. When the SS content increases from 20% to 40%, the compressive strength of the specimens markedly improves, demonstrating that an appropriate amount of SS provides a more favorable alkaline environment, which promotes precursor dissolution and subsequent hydration reactions, accelerates gel product formation, and improves the pore structure and overall compactness of the matrix; consequently, the material exhibits higher compressive performance. Among all mixtures, the M40 group achieves the highest 28-day compressive strength of 38.7 MPa, which is approximately 24.4%, 9.3%, and 39.7% higher than those of the M20, M60, and M80 groups, respectively, further confirming that at this dosage the system achieves the optimal degree of reaction and microstructural improvement. However, when the SS content is further increased to 60% and 80%, the compressive strength of the specimens decreases instead. This decline is mainly attributed to the excessively high SS content significantly accelerating the early reaction kinetics of the system, leading to rapid encapsulation of FA particle surfaces by quickly formed reaction products. This encapsulation inhibits further dissolution and sustained reaction, reduces the overall reaction degree and the amount of gel formed at later ages, hinders continuous densification of the structure, and ultimately results in decreased compressive strength. Notably, for the M80 group, although the strength increase from 7 to 14 days is relatively pronounced, the strength gain from 14 to 28 days is substantially weakened, indicating that under high SS content conditions, the late-age strength development of the system is limited.

**Figure 7:**
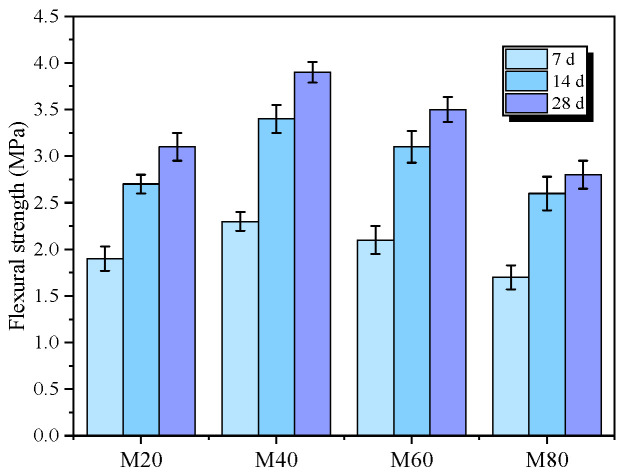
Flexural strength of phosphogypsum–steel slag–fly ash cementitious material.

Figure 7 presents the flexural strength development of PSA with different SS contents. As shown in Figure 7, the flexural strength generally increased first and then decreased with increasing SS content, and this trend was consistent at all curing ages. Among all mixtures, M40 achieved the highest 28 d flexural strength of 3.9 MPa, representing an increase of 25.8% compared with M20. Moreover, the evolution of flexural strength was broadly consistent with that of compressive strength, indicating that an appropriate incorporation of SS can effectively enhance the mechanical performance of PSA. The strengthening effect was particularly pronounced when the SS content was in the range of 20–40%.

### 3.4. Hydration Products and Hydration Process

#### 3.4.1. X-Ray Diffraction Analysis

**Figure 8:**
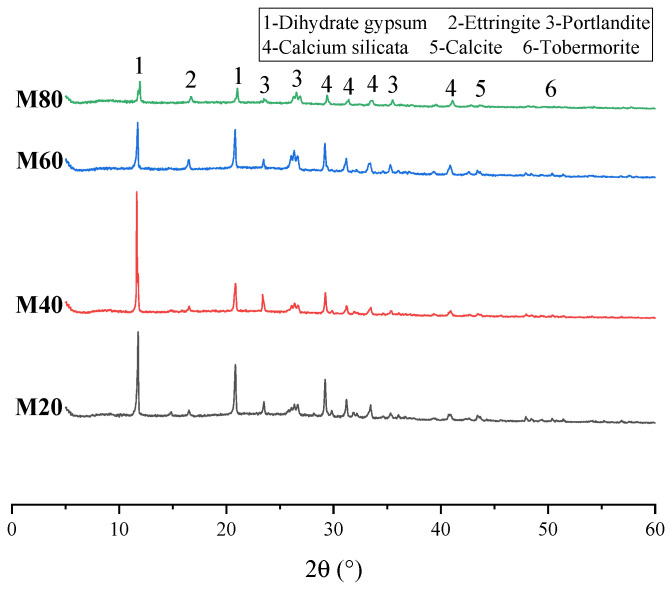
X-ray diffraction patterns of phosphogypsum–steel slag–fly ash cementitious material.

Figure 8 shows the XRD patterns of PSA with different SS contents. The results indicate that the phase assemblages of all mixtures were essentially similar, suggesting that variations in SS dosage did not change the types of hydration products formed. The main crystalline phases identified were gypsum, ettringite, calcium hydroxide, calcium silicate, tobermorte, and calcite. Compared with M20, M40 exhibited more pronounced characteristic diffraction peaks in the low-angle region, together with a clear increase in the intensities of several major crystalline phases, indicating that appropriate SS incorporation promoted the dissolution of reactive Ca-, Al-, and Si-bearing species and their subsequent reactions. As a result, the formation of crystalline products such as gypsum and ettringite became more sufficient, which was beneficial for the development of a denser internal structure. When the SS content was further increased to 60%, M60 still retained relatively distinct characteristic peaks, indicating a comparatively high reaction degree; however, the peak distribution became more dispersed, reflecting more pronounced coexistence of multiple phases within the system. In contrast, M80 showed a marked reduction in overall diffraction intensity, suggesting that an excessively high SS dosage was unfavorable for the sustained formation of effective reaction products, thereby leading to a lower content of crystalline phases and/or reduced structural ordering. In addition, calcite peaks were detected in all mixtures, implying that different degrees of carbonation occurred during curing or sample preparation. Overall, the SS content played a significant role in regulating the phase evolution of PSA, and an appropriate SS dosage promoted the formation of crystalline products and increased the overall reaction extent.

#### 3.4.2. Thermogravimetric and Derivative Thermogravimetric Analysis

**Figure 9:**
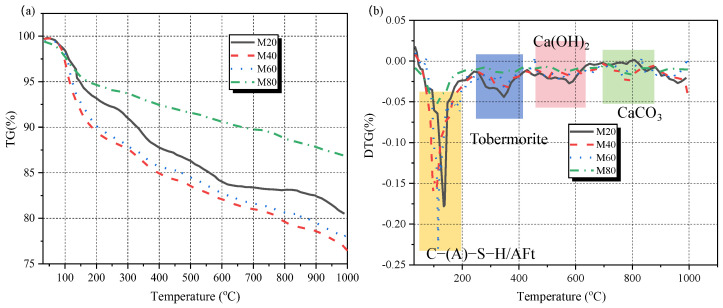
Thermogravimetric and derivative thermogravimetric curves of phosphogypsum–steel slag–fly ash cementitious material: (**a**) thermogravimetric (TG) curves; (**b**) derivative thermogravimetric (DTG) curves.

Figure 9 presents the TG–DTG curves of PSA. All mixtures exhibited four major mass loss peaks in the DTG curves, located at 100–200 °C, 200–300 °C, 400–500 °C, and 600–800 °C, respectively. The mass loss in the range of 100–200 °C was mainly associated with the removal of water from C-(A)-S-H gel and AFt [36]. The mass loss between 200 and 300 °C was attributed to the loss of structural water from tobermorite [37]. The peak observed at 400–500 °C corresponded to the dehydroxylation of Ca(OH)_2_, while the mass loss in the range of 600–800 °C was assigned to the decomposition of CaCO_3_ [36,38]. This result is consistent with the detection of calcite in the XRD patterns, further confirming the presence of a certain amount of carbonate products in the samples.

Table 3 summarizes the phase-related and total mass losses of PSA with different SS contents during heating. The total mass loss first increased and then decreased with increasing SS content, reaching a maximum in M40 (23.6%), followed by M60 (21.9%) and M20 (19.5%), while M80 showed the lowest value (13.2%). This indicates that a moderate SS dosage promoted the overall reaction and increased the amount of hydration products, whereas excessive SS dosage reduced the reaction extent. The mass loss associated with C-(A)-S-H/AFt followed the order of M40 > M60 > M20 > M80, with M40 and M60 showing markedly higher values, confirming that appropriate SS incorporation favored gel and AFt formation. In contrast, the mass loss assigned to tobermorite varied only slightly among M20-M60 but decreased substantially in M80, suggesting that excessive SS hindered the formation and stability of calcium-silicate-type products. Meanwhile, the gradual decrease in Ca(OH)_2_-related mass loss with increasing SS content, together with the higher gel-related mass loss in M40 and M60, implies that CH was further consumed in subsequent reactions. The CaCO_3_-related mass loss generally followed the same trend as the calcite peak intensity in XRD, confirming the occurrence of carbonation in all mixtures. Overall, M40 exhibited the highest total and gel-related mass losses, indicating the highest reaction degree and the greatest amount of effective reaction products.

#### 3.4.3. FTIR

**Figure 10:**
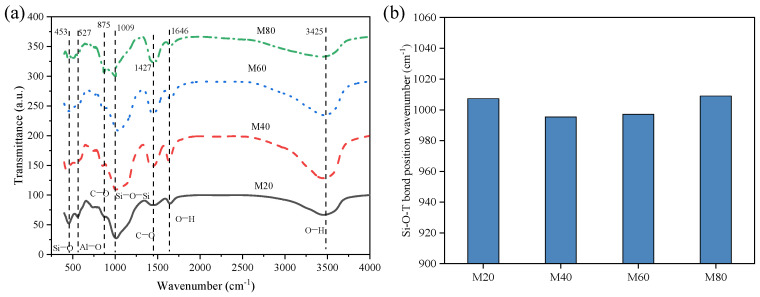
(**a**) Fourier transform infrared spectroscopy spectra of PSA; (**b**) Si–O–T bond wavenumber variation of samples.

Figure 10 presents the FTIR spectra of PSA specimens cured for 28 days with different SS contents. As can be seen from Figure 10a, the absorption band at 3450 cm^−1^ corresponds to the O–H stretching vibration of water molecules, while the band at 1640 cm^−1^ is attributed to the H–O–H bending vibration [39]. The characteristic bands at 1463 cm^−1^ and 875 cm^−1^ correspond to different vibration modes of CO_3_^2−^ [40], indicating the presence of carbonate products in the samples, which is consistent with the detection of calcite by TG and XRD. The absorption bands at 453 cm^−1^ and 627 cm^−1^ are assigned to the stretching vibrations of Si–O and Al–O bonds in the raw materials, respectively [41]. Furthermore, the broad absorption band in the range of 1100–950 cm^−1^ is associated with the asymmetric stretching vibration of Si–O–T (T = Si or Al) bonds [42]; this band is generally considered an important indicator of the formation of an aluminosilicate gel structure, reflecting the generation of hydration products and the reconstruction of the silicon–aluminum framework in the system.

The characteristic absorption band associated with the Si–O–T bridging bond (T = Si or Al) reflects the polymerization and structural evolution of the aluminosilicate network in the PSA system. As shown in Figure 9b, as the SS content increases from 20% to 80%, the wavenumber of the characteristic peak corresponding to the Si–O–T bond in the reaction products first decreases and then increases, with the M40 group exhibiting the lowest peak position at 995.4 cm^−1^. This result indicates that with an appropriate SS dosage, more Al atoms are incorporated into the C-(A)-S-H gel skeleton, substituting some Si sites, thereby promoting a higher degree of crosslinking within the gel network. This observation is consistent with the findings reported in the literature [43]. When the SS content is further increased from 40% to 80%, the Si–O–T characteristic peak shifts from 995.4 cm^−1^ to 1009 cm^−1^, suggesting a reduced degree of Al incorporation into the gel and a weakened reconstruction of the silicon–aluminum framework. This is primarily attributed to the excessively high SS content suppressing the effective reaction of the system, resulting in insufficient dissolution of Al, which in turn limits its ability to participate in the structural evolution of the C-(A)-S-H gel.

#### 3.4.4. SEM

**Figure 11:**
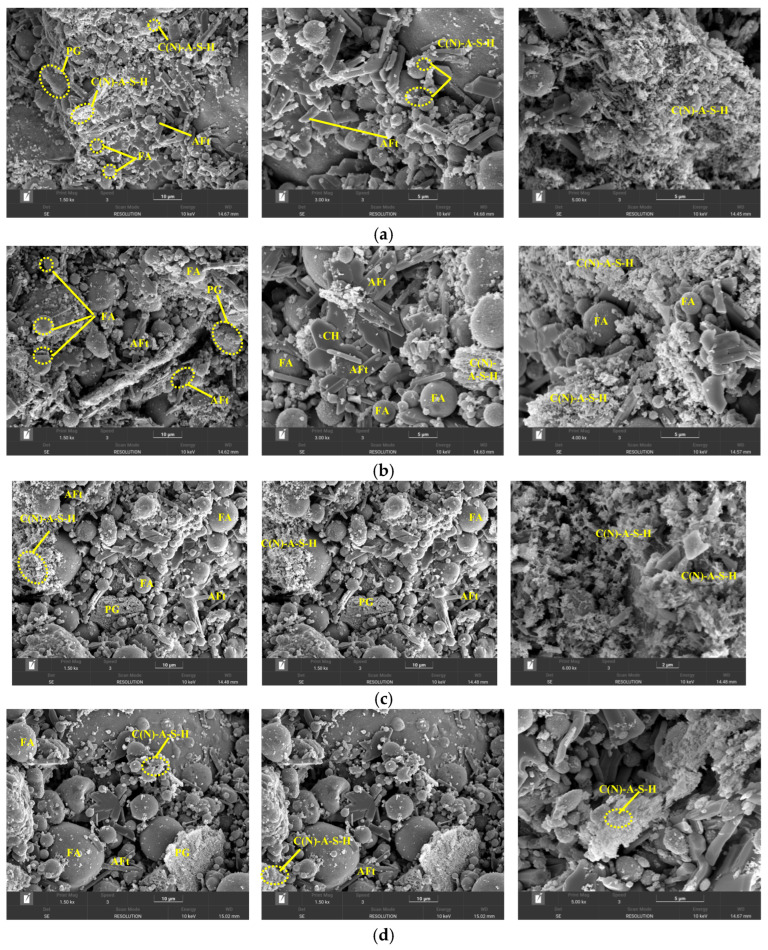
SEM of PSA. (**a**) M20; (**b**) M40; (**c**) M60; (**d**) M80.

To further elucidate the hydration process and microstructural evolution of the PSA system, SEM was employed to characterize the morphologies of samples with different SS contents after curing, as shown in Figure 11. Distinct microstructural differences were observed among the mixtures. In M20 (Figure 11a), a large amount of needle-like AFt, unreacted PG and FA particles, and flocculent C–(A)–S–H gel can be clearly identified. The flocculent gel adhered to and precipitated on the surfaces of the irregular PG and FA particles while also filling the skeletal pores formed by PG particles in the form of clusters, thereby contributing to matrix densification. Meanwhile, the needle-like AFt crystals were interwoven and widely distributed within the cracks and pores of the matrix, forming a three-dimensional network structure. Some AFt crystals were also observed to grow around the surfaces of PG particles and bond tightly to them, resulting in relatively indistinct interfacial boundaries. When the SS content increased to 40% (Figure 11b), AFt crystals remained widely distributed in the pores and cracks, but their cluster density became markedly higher than that in M20. In addition, the amount of AFt attached to the surfaces of unreacted PG and FA particles increased significantly, indicating that a moderate SS dosage effectively promoted AFt formation, which is consistent with the TG–DTG results. At the same time, the amount of flocculent gel on particle surfaces also increased noticeably, and more gel clusters filled the internal pores of the particle skeleton, leading to a denser and more integrated microstructure. These observations confirm that an appropriate increase in SS content can enhance the overall reaction degree and improve matrix compactness.

When the SS content was further increased to 60% (Figure 11c), the density of AFt crystals decreased to some extent, accompanied by a reduction in flocculent gel content and a lower crystal aspect ratio. However, the overall variation remained relatively limited, suggesting that this mixture still maintained a comparatively high reaction degree. In contrast, when the SS content reached 80% (Figure 11d), the amount of unreacted PG and FA particles increased markedly, whereas the quantities of AFt and flocculent gel decreased substantially. As a result, more loose regions and pore defects appeared within the matrix, and the overall compactness was significantly reduced. This indicates that an excessively high SS dosage inhibited the nucleation and growth of AFt and gel phases, thereby weakening the effective reaction extent and deteriorating the microstructure, in agreement with previous findings in the literature [35,44]. Overall, the SEM observations demonstrate that a moderate SS dosage is beneficial for promoting the formation of AFt and C-(A)-S-H gel and for enhancing matrix compactness through pore filling and the development of a more continuous microstructure. In contrast, excessive SS hinders the sustained formation of reaction products and is therefore unfavorable for microstructural optimization. These microstructural features are consistent with the pore structure and mechanical results, further confirming that the superior performance of M40 is closely associated with its denser and more homogeneous internal structure.

### 3.5. Microstructure

**Figure 12:**
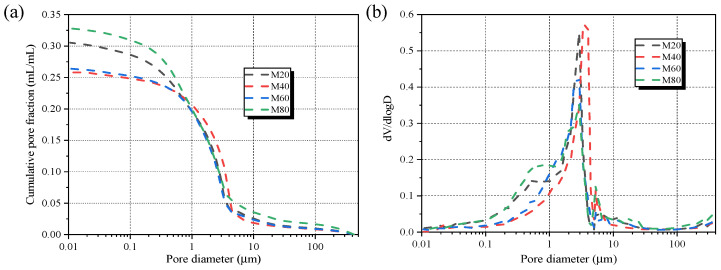
Pore structure characteristics of PSA: (**a**) cumulative pore volume; (**b**) pore size distribution.

Figure 12 shows the cumulative porosity and pore size distribution of PSA with different SS contents. For all mixtures, the pore size was mainly concentrated in the range of 1–10 µm. With increasing SS content, the total porosity first decreased and then increased, with the lowest value being observed for M40 (25.8%). Compared with M20, the total porosity of M40 and M60 decreased by 15.7% and 13.4%, respectively, indicating that appropriate SS incorporation can effectively refine the pore structure and enhance matrix compactness, with the most pronounced densification occurring at an SS content of 40%. However, when the SS content was further increased, this beneficial effect became weaker, and the porosity of M80 instead increased by 7.5%.

To further clarify the pore refinement effect, the pores were classified into three categories according to the method proposed by Zheng [45], namely small pores (<50 nm), medium pores (50–100 nm), and large pores (>100 nm), as shown in Table 4. In all mixtures, large pores overwhelmingly dominated the pore system, accounting for 93.10–95.29% of the total porosity, indicating that the overall porosity of PSA was primarily governed by the large pore fraction. The large pore contents of M20, M40, M60, and M80 were 29.16%, 24.51%, 25.17%, and 30.63%, respectively, and their variation was highly consistent with that of total porosity. This result demonstrates that the reduction in total porosity was mainly associated with the marked decrease in large pores. In particular, M40 exhibited the most pronounced reduction in the large pore fraction, confirming that this SS dosage was the most effective in promoting a denser and more homogeneous matrix. In contrast, the increase in all pore classes in M80 indicates that excessive SS not only weakened the pore refinement effect but also induced structural loosening. From a mechanistic perspective, an appropriate SS dosage is beneficial for establishing a more favorable reaction environment, which promotes precursor dissolution, gel formation, and pore filling, thereby suppressing the development of coarse pores and improving matrix densification. In contrast, excessive SS may disturb the coordination of the reaction process and restrict the sustained development of later-age reactions, resulting in insufficient pore filling and deterioration of the pore structure. These pore structure characteristics are consistent with the mechanical strength results, further confirming that the superior mechanical performance of M40 is closely associated with its more compact microstructure and lower fraction of large pores.

## 4. Discussion

Unlike conventional cement-based systems, where solid wastes are generally used only as supplementary cementitious materials or inert fillers, the performance evolution of PSA is governed by synergistic interaction among PG, SS, and FA as multiple reactive constituents. In this ternary all-solid-waste system, PG mainly provides SO_4_^2−^ and part of the available Ca^2+^, steel slag supplies both alkalinity and reactive Ca through hydration and dissolution, and FA serves as the principal source of Si- and Al-bearing species for subsequent gel formation. Accordingly, the hardening and strength development of PSA do not originate from a single reaction pathway but from the coupled effects of sulfate activation, alkaline activation, and pozzolanic reaction. The balance among these processes determines the formation of AFt and C-(A)-S-H products, the evolution of pore structure, and, ultimately, the mechanical performance of the system. Therefore, to clarify the governing role of SS in regulating the reaction process and structure development of PSA, the compressive and flexural strengths, pore characteristics, phase assemblage, thermal behavior, FTIR, and microstructural features are discussed in the following sections based on the results presented in Figure 5, Figure 6, Figure 7, Figure 8, Figure 9, Figure 10, Figure 11 and Figure 12.

CaO + H_2_O → Ca(OH)_2_ (1)

Ca(OH)_2_ ⇌ Ca^2+^ + 2OH^−^ (2)

CaSO_4_·2H_2_O → Ca^2+^ + SO_4_^2−^ + 2H_2_O (3)

(Si,Al)-glass + OH^−^ + H_2_O → [SiO(OH)_3_]^−^ + Al(OH)_4_^−^ (4)

6Ca^2+^ + 2Al(OH)_4_^−^ + 3SO_4_^2−^ + 4OH^−^ + 26H_2_O → Ca_6_Al_2_(SO_4_)_3_(OH)_12_·26H_2_O (5)

Ca^2+^ + [SiO(OH)_3_]^−^ + Al(OH)_4_^−^ + H_2_O → C-(A)-S-H (6)

**Figure 13:**
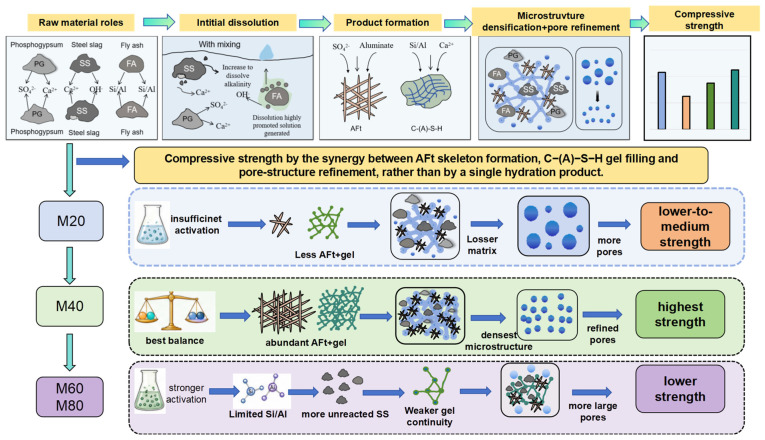
Schematic diagram of reaction mechanism of PSA.

Based on the above results, the evolution of strength, pore structure, phase assemblage, and microstructure in the PSA system can be interpreted in terms of the coupled reaction pathways schematically illustrated in Figure 13. The reaction of PSA is initiated by the combined effects of alkaline activation from SS and sulfate supply from PG. Specifically, the hydration and dissolution of reactive Ca-bearing phases in SS release Ca^2+^ and OH^−^ (Equations (1) and (2)), thereby increasing the alkalinity of the pore solution and promoting the depolymerization and dissolution of the vitreous aluminosilicate phases in FA, which leads to the continuous release of reactive Si- and Al-bearing species into the liquid phase (Equation (4)). Meanwhile, the dissolution of PG provides SO_4_^2−^ together with part of the Ca^2+^ (Equation (3)); these species subsequently react with dissolved aluminate species to form AFt (Equation (5)). As the reaction proceeds, the dissolved Si and Al species further interact with Ca^2+^ through precipitation and polycondensation reactions to generate C-(A)-S-H gel (Equation (6)). Therefore, the hardening of PSA is governed not by a single hydration process but by the synergistic coupling of sulfate activation, alkaline activation, and pozzolanic reaction. This interpretation is consistent with the XRD results, which indicate that AFt, C-(A)-S-H gel, residual PG, and a small amount of carbonate phases are the main reaction products, confirming the coexistence of sulfate-driven crystallization and aluminosilicate gelation in the system. More importantly, the balance among these reactions is strongly dependent on SS dosage. At an appropriate SS content, the enhanced alkaline environment facilitates precursor dissolution, promotes the formation of AFt and gel products, and improves pore filling and matrix densification, thereby resulting in superior mechanical performance. In contrast, excessive SS disturbs the coordination of the reaction process, restricts the sustained dissolution of FA and the later-stage formation of effective products, and ultimately leads to microstructural deterioration and strength reduction.

Combined with the FTIR and TG–DTG results, it is evident that increasing the SS content from 20% to 40% significantly promoted the formation of AFt and C-(A)-S-H gel. The enhanced bound-water loss and stronger decomposition peaks in TG–DTG indicate an increased reaction degree, while the shift of the Si–O–T band toward lower wavenumbers in FTIR suggests that more Al released from FA dissolution was incorporated into the aluminosilicate gel network, leading to a higher degree of gel polymerization. These results indicate that within the 20–40% range, the increase in SS effectively enhanced the alkalinity and reactive Ca supply of the system, thereby promoting FA dissolution, PG consumption, and the coupled formation of AFt and gel products. This range can therefore be regarded as the effective activation interval of SS in which its alkali-providing and Ca-supplying functions played a dominant role in enhancing the overall reaction.

At 40% SS, the formation of AFt crystals and the accumulation of C-(A)-S-H gel reached the most favorable balance. SEM observations show that needle-like AFt and flocculent gel were interwoven and jointly filled the pore space in M40, resulting in the most compact microstructure. This is consistent with the MIP results, which reveal a marked reduction in both total porosity and the large pore fraction for M40. These findings suggest that the skeleton-forming effect of AFt and the pore-filling effect of the gel phase acted synergistically to refine the pore structure and improve matrix compactness. Consequently, the superior performance of M40 can be attributed to the most favorable balance between sulfate supply, alkaline activation, and gel formation, which enables the development of a denser and more homogeneous internal structure.

However, when the SS content was further increased to 60% and 80%, the increase in effective hydration products became less pronounced and even showed a declining trend. Although higher SS contents could still provide a stronger alkaline environment and a greater supply of Ca^2+^, the corresponding reduction in FA content limited the continuous release of reactive Si- and Al-bearing species, thereby restricting the later-stage formation of C-(A)-S-H gel. At the same time, some low-reactivity minerals in excessive SS could not effectively participate in the reaction and thus remained in the matrix as unreacted particles, generating local interfacial defects and weakening the continuity of the gel network. This interpretation is supported by the SEM observations, in which M60 and M80 exhibited reduced gel coverage, weakened pore-filling effects, and a higher amount of unreacted particles. Consistently, the MIP results showed that the pore refinement effect was weakened in these two mixtures, accompanied by an increase in the proportion of large pores. These findings indicate that excessive SS disrupted the synergistic balance between hydration product formation and pore filling, ultimately resulting in reduced matrix compactness and lower compressive strength.

Overall, the performance enhancement of the PSA ternary all-solid-waste system does not depend on an increase in any single hydration product alone. At an appropriate SS dosage, favorable synergy is established among the AFt crystal framework, C-(A)-S-H gel filling, and pore structure refinement, which significantly enhances the reaction degree and mechanical performance of the system. In contrast, when the SS content becomes excessively high, the insufficient supply of reactive aluminosilicate species together with the retention of unreacted SS particles destroys this synergy, leading to a looser microstructure and consequent strength deterioration.

In addition, it should be noted that the residual soluble P and F impurities in PG may delay early-age reactions, while the free CaO and MgO in SS may also pose potential risks to volumetric stability. Therefore, the application of a full solid waste binder system requires not only attention to strength development but also a comprehensive evaluation incorporating expansion rate, drying shrinkage, softening coefficient, heavy metal leaching, and long-term durability. Moreover, the present study mainly evaluated the mechanical properties of PSA mortars at 7, 14, and 28 d, while the very early-age strength development before 7 days was not systematically investigated. Therefore, although the optimized M40 mixture exhibited the highest 28-day compressive strength and the densest microstructure, the application of this modified cementitious binder in concrete structures requiring rapid demolding, early loading, or accelerated construction should be treated with caution. The relatively slow early reaction of the fly-ash-rich system and the dependence on the gradual formation of AFt and C-(A)-S-H gel may limit its suitability for early-strength concrete. For practical use, this binder is more suitable for applications where later-age strength development and solid waste utilization are prioritized over very early strength. Future studies should further evaluate the 1-day and 3-day strength of PSA concrete and explore early-strength enhancement strategies, such as optimized curing regimes, particle size refinement, activator adjustment, or the incorporation of suitable accelerators.

## 5. Conclusions

This study systematically investigates the PG-SS-FA ternary cementitious system (PSA) with varying SS dosages (20–80%). The setting time, fluidity, compressive strength, and flexural strength of the composite binder are comprehensively evaluated. The main findings are as follows:

(1) With increasing SS content, the setting time of the PSA system first shortened and then prolonged, whereas the fluidity continuously decreased. The fastest setting behavior was observed at an SS content of 40%, with initial and final setting times of 126 and 321 min, respectively. This indicates that an appropriate SS dosage accelerates the hardening process of PSA, while excessive SS weakens this promoting effect.
(2) As the SS content increased from 20% to 40%, the hydration reaction of the system was significantly enhanced, as evidenced by the highest amounts of AFt and C-(A)-S-H formation and refinement of the pore structure. Among all mixtures, M40 exhibited the highest reaction degree and the most pronounced structural densification. However, when the SS content was further increased to 60% and 80%, the hydration reaction was weakened. This was mainly because excessive SS, despite increasing the Ca^2+^ supply and alkalinity, reduced the sustained availability of reactive Si/Al species from FA, ultimately limiting later-age gel accumulation and pore structure refinement.
(3) The performance evolution of the PSA system is governed by the coupled effects of alkali and calcium supply from SS, sulfate supply from PG, and reactive Si/Al supply from FA. Strength development mainly arises from the synergistic contribution of the AFt crystal framework and the continuous pore-filling effect of C-(A)-S-H gel.
(4) With increasing SS content, both the compressive and flexural strengths of PSA first increased and then decreased. The highest compressive and flexural strengths were obtained at an SS content of 40%, which was closely associated with the greatest formation of AFt and C-(A)-S-H gel, as well as the most pronounced pore structure refinement.

## Figures and Tables

**Table 1:** Chemical compositions of precursor (%).

Material Types	CaO	SiO_2_	Al_2_O_3_	Fe_2_O_3_	MgO	Na_2_O	SO_3_	Others
SS	56.82	27.95	2.35	3.41	4.26	0.44	0.94	3.83
FA	5.48	52.35	28.68	4.57	1.14	1.15	1.48	5.15
PG	38.76	2.20	0.29	1.15	0.21	0.14	56.01	1.25
OPC	53.44	24.78	4.26	3.88	3.06	0.32	4.27	5.99

Notes: The chemical composition of OPC is sourced from reference [30].

**Table 2:** Mixture proportions of samples (wt/%).

Group	SS (%)	FA (%)	PG (%)
M20	20	53.3	26.7
M40	40	40	20
M60	60	26.7	13.3
M80	80	13.3	6.7

**Table 3:** Mass loss of main phases in PSA (%).

Group	C-(A)-S-H/AFt	Tobermorite	Ca(OH)_2_	Calcite	Total Mass Loss
M20	7.1	2.2	1.5	1.5	19.5
M40	10.6	2	1.4	2.8	23.6
M60	9.9	2.2	1.2	2.2	21.9
M80	5.4	0.9	0.9	1.8	13.2

**Table 4:** Pore structure of PSA.

Group	Cumulative Porosity (%)	Pore Size Distribution (%)		
		<50 nm	50–100 nm	>100 nm
M20	30.6	1.20	0.24	29.16
M40	25.8	1.06	0.23	24.51
M60	26.5	1.11	0.22	25.17
M80	32.9	1.95	0.32	30.63

## Data Availability

The original contributions presented in this study are included in the article. Further inquiries can be directed to the corresponding author.

## Abbreviations

The following abbreviations are used in this manuscript:

OPC	Ordinary Portland cement
PG	Phosphogypsum
SS	Steel slag
FA	Fly ash
PSA	PG-SS-FA cementitious material
C-(A)-S-H	Calcium–aluminosilicate–hydrate
XRD	X-ray diffraction
TG-DTG	Thermogravimetric analysis
FTIR	Fourier transform infrared spectroscopy
MIP	X-ray fluorescence
SEM	Scanning electron microscopy
